# PD84 Impact Of Public Debate On Health Technology Assessment Based Decisions: Brazilian Case Of Left Atrial Appendage Closure In The Supplementary Health Sector

**DOI:** 10.1017/S0266462325102110

**Published:** 2025-12-29

**Authors:** Maiara Antonio, Henrique Tortele, Anna Pereira, Murilo Contó

## Abstract

**Introduction:**

Atrial fibrillation (AF) has a high economic burden for the healthcare system. Due to the emboligenic profile of AF, stroke prevention with oral anticoagulants (OAC) is usually recommended. For patients with AF at risk of bleeding who are contraindicated for OAC, non-pharmacological thromboembolism prevention therapies such as left atrial appendage closure (LAAC) is recommended. We aimed to incorporate the LAAC procedure into the Brazilian Supplementary Health Sector (SHS).

**Methods:**

A scientific technical opinion (STO) including a literature review and economic and budget impact analysis was submitted to the National Supplementary Health Agency (ANS). The STO was technically evaluated and a health technology assessment based ANS decision rite was followed. The rite was composed of an applicant defense meeting, publication of an ANS critical report, a preliminary decision, public consultation, a public hearing, presentation of the public consultation results, and a final decision. Applicants could participate in the defense meeting and all stages of public participation.

**Results:**

Arguing insufficient randomized controlled trials specifically addressing the OAC-contraindicated AF population and some concerns regarding the economic modeling, ANS preliminary decision was unfavorable to the incorporation. The ANS full critical report was published and received extensive responses from the applicants in the public consultation, which received 919 contributions. The main points addressed during public debates, including the public hearing were the ethical implications of allocating OAC-contraindicated patients to an OAC treatment arm; the lack of treatment available for this population in the Brazilian SHS; the importance of real-world evidence; the impact of stroke on patients, families, and society; and clarifications on the economic model. After considering all the contributions, the ANS reversed its verdict.

**Conclusions:**

The LAAC procedure has been available in the Brazilian SHS since 1 July 2024. The ANS’ final favorable decision was only possible thanks to the broad space for public debate made available during the ANS decision rite, added to extensive popular engagement throughout the process.

